# Effects of acute sleep deprivation on individuals across different *APOE* genotypes: a randomized controlled trial protocol

**DOI:** 10.3389/fneur.2026.1805467

**Published:** 2026-09-22

**Authors:** He-Jin Wang, Ling-Ling Liu, Yi-Hang Li, Yu-Hui Qiu, Xue Xia, Ye-Feng Cai, Shi-Jie Zhang

**Affiliations:** 1 Department of Neurology, The Second Affiliated Hospital of Guangzhou University of Chinese Medicine, Guangzhou, Guangdong, China; 2 Department of Neurology, Guangdong Provincial Hospital of Chinese Medicine, Guangzhou, Guangdong, China; 3 State Key Laboratory of Traditional Chinese Medicine Syndrome, The Second Affiliated Hospital of Guangzhou University of Chinese Medicine, Guangzhou, Guangdong, China; 4 Department of Neurology, Beijing Tiantan Hospital, Capital Medical University, Beijing, China

**Keywords:** Alzheimer’s disease, APOE, biomarkers, multi-omics, sleep deprivation

## Abstract

**Background:**

Sleep is crucial for the clearance of metabolic waste products. Sleep deprivation leads to the accumulation of amyloid-*β* (Aβ) and tau proteins, which may be mediated by impaired glymphatic clearance, thereby contributing to the hallmark pathologies of Alzheimer’s disease (AD). The apolipoprotein E (APOE) gene, particularly the *APOE* ε4 allele, is a major genetic risk factor for AD and is also closely associated with sleep. Notably, the interaction between poor sleep and APOE ε4 may further enhance AD risk. However, prior studies have reported inconsistent effects of sleep deprivation on AD related biomarkers, and clinical studies stratified by *APOE* genotype are lacking. This study aims to investigate the effects of 24-h acute sleep deprivation on plasma AD biomarkers and to assess *APOE* genotype-dependent responses.

**Methods:**

This prospective, randomized open-label, blinded-endpoint trial plans to enroll 60 healthy young adults. Participants will be stratified by *APOE* genotype into three groups at a 1:1:1 ratio: *APOE* ε3/ε3 homozygotes, *APOE* ε4 carriers (ε3/ε4 and ε4/ε4 genotypes), and *APOE* ε2 carriers (ε2/ε3 and ε2/ε2 genotypes). Within each genotype stratum, participants will be randomly assigned in a 1:1 ratio to either the 24-h acute sleep deprivation group or the normal sleep group. The primary outcome is the change in plasma Aβ_42_ levels from baseline to day 1 after randomization. Secondary outcomes include changes from baseline to day 1 after randomization in plasma levels of the following biomarkers: Aβ_40_, total tau (t-tau), phosphorylated tau-181 (p-tau181), phosphorylated tau-217 (p-tau217), neurofilament light chain (NfL), and glial fibrillary acidic protein (GFAP). Additionally, the following ratios will be assessed: Aβ_42_/Aβ_40_, t-tau/Aβ_42_, p-tau181/Aβ_42_, and p-tau217/Aβ_42_. Changes in neuropsychiatric and cognitive assessment will also be evaluated, including scores on the Fatigue Scale-14 (FS-14), the Positive and Negative Affect Schedule (PANAS), and performance on the N-back tasks. Exploratory outcomes include changes in plasma proteomics and metabolomics, as well as positron emission tomography/magnetic resonance imaging (PET/MRI) measures.

**Discussion:**

This study serves as the first randomized controlled trial (RCT) to explore the potential influence of acute sleep deprivation on plasma AD biomarkers across different *APOE* genotypes, with the largest sample size among current RCTs on sleep deprivation. This trial is expected to generate critical evidence elucidating the impact of 24-h acute sleep deprivation on the pathophysiology of AD and to inform the development of future sleep-focused preventive interventions.

**Study protocol registration:**

ClinicalTrials.gov, identifier NCT07085754.

## 1 Background

Sleep is an essential component of human life and a physiological process that plays a crucial role in maintaining human health. Its restorative function helps to clear metabolic waste products (1) that build up in the brain while we are awake (2). Studies on animals demonstrate that sleep increases the proportion of interstitial fluid in the brain by 60%, thereby doubling the clearance rate of amyloid-*β* (Aβ) in the cortex (3). In healthy individuals, one night of free sleep can clear 6% of Aβ_42_ protein from cerebrospinal fluid (CSF) (4). Aβ and other metabolic waste products such as tau protein constitute the primary pathological hallmarks of Alzheimer’s disease (AD) (5), whilst sleep deprivation may lead to the accumulation of such metabolic waste.

Sleep deprivation is a state of consistently insufficient sleep relative to one’s biological needs, resulting from voluntary choices, medical conditions, or environmental factors (6). Sleep deprivation is commonly seen in professions such as doctors and night shift employees (7, 8). Approximately one-third of the global population is affected by sleep deprivation (7). A study measuring adult cerebrospinal fluid (CSF) demonstrated that subjects who underwent 36 h of sleep deprivation had a 51.5% higher tau level compared to those with normal sleep (9). Meanwhile, evidence indicates that Aβ burden increase specifically in the right hippocampus and thalamus following one night of total sleep deprivation (10). In a crossover study of cognitively normal adults, CSF concentrations of Aβ_40_, Aβ_42_, and tau species (including unphosphorylated tau at threonine 181, unphosphorylated tau at threonine 217 and phosphorylated tau at T181) increased by approximately 35–55% during sleep deprivation, while corresponding plasma concentrations decreased by about 5–15%, resulting in elevated CSF-to-plasma ratios (11). A growing number of plasma AD biomarkers exhibit strong correlations with AD pathology (11, 12). However, previous studies have produced inconclusive results regarding the effects of sleep deprivation on plasma AD biomarker levels, and it remains unclear whether these effects depend on genetic factors.

Apolipoprotein E (*APOE*) is a genetic susceptibility factor for AD, particularly the *APOE* ε4 allele. The *APOE* gene has three allelic variants: *APOE* ε2*, APOE* ε3*, and APOE* ε4. Studies have shown that the *APOE* ε2 allele is associated with reduced cerebral Aβ deposition in both cognitively normal elderly individuals and patients with AD (13–17). In contrast, *APOE* ε4 carriers face a significantly higher risk of developing AD compared to the most common genotype, *APOE* ε3 homozygotes (18). *APOE* is also linked to sleep disorders. Previous research has shown that *APOE* ε4 is associated with sleep disorders in the elderly (19), as well as an increased risk of insomnia (20) and obstructive sleep apnea (21, 22) in adults. An animal study indicated that *APOE* ε4 may act synergistically with sleep deprivation to accelerate AD-related pathological changes (23). However, there are currently no clinical studies investigating the effects of sleep deprivation on AD biomarker levels in populations with different *APOE* genotypes.

Therefore, this study is designed to assess the effects of a 24-h acute sleep deprivation on plasma AD biomarkers and to examine potential differences in these effects across *APOE* genotypes. Furthermore, this study will characterize concomitant neuroimaging alterations and apply a multi-omics approach to identify associated changes in protein and metabolite expression. This integrated strategy aims to elucidate the underlying mechanisms and molecular signatures of acute sleep deprivation, thereby establishing a foundation for future research and potential translational applications.

## 2 Methods

### 2.1 Design and setting

This prospective, randomized open-label, blinded-endpoint trial will be conducted at Guangdong Provincial Hospital of Chinese Medicine. A total of 60 healthy participants will be enrolled in a 1:1:1 ratio to three *APOE* genotypes groups: *APOE* ε3/ε3 homozygotes, *APOE* ε4 carriers (ε3/ε4 and ε4/ε4 genotypes), and *APOE* ε2 carriers (ε2/ε3 and ε2/ε2 genotypes). Given the potential opposing effects of the ε2 and ε4 alleles on Alzheimer’s disease risk, participants with the *APOE* ε2/ε4 genotype will be excluded. Within each genotype stratum, participants will be randomly assigned in a 1:1 ratio to one of two groups: an intervention group undergoing 24-h acute sleep deprivation or the control group maintaining normal sleep. Figure 1 shows the flowchart of the trial, and Table 1 outlines the study timeline. This study protocol complies with the Declaration of Helsinki and has been approved by the Ethics Committee of Guangdong Provincial Hospital of Chinese Medicine (ZF-2025-206-01) and was registered on the ClinicalTrials.gov platform (NCT07085754). The trial protocol will be available at ClinicalTrials.gov.

**Figure 1:**
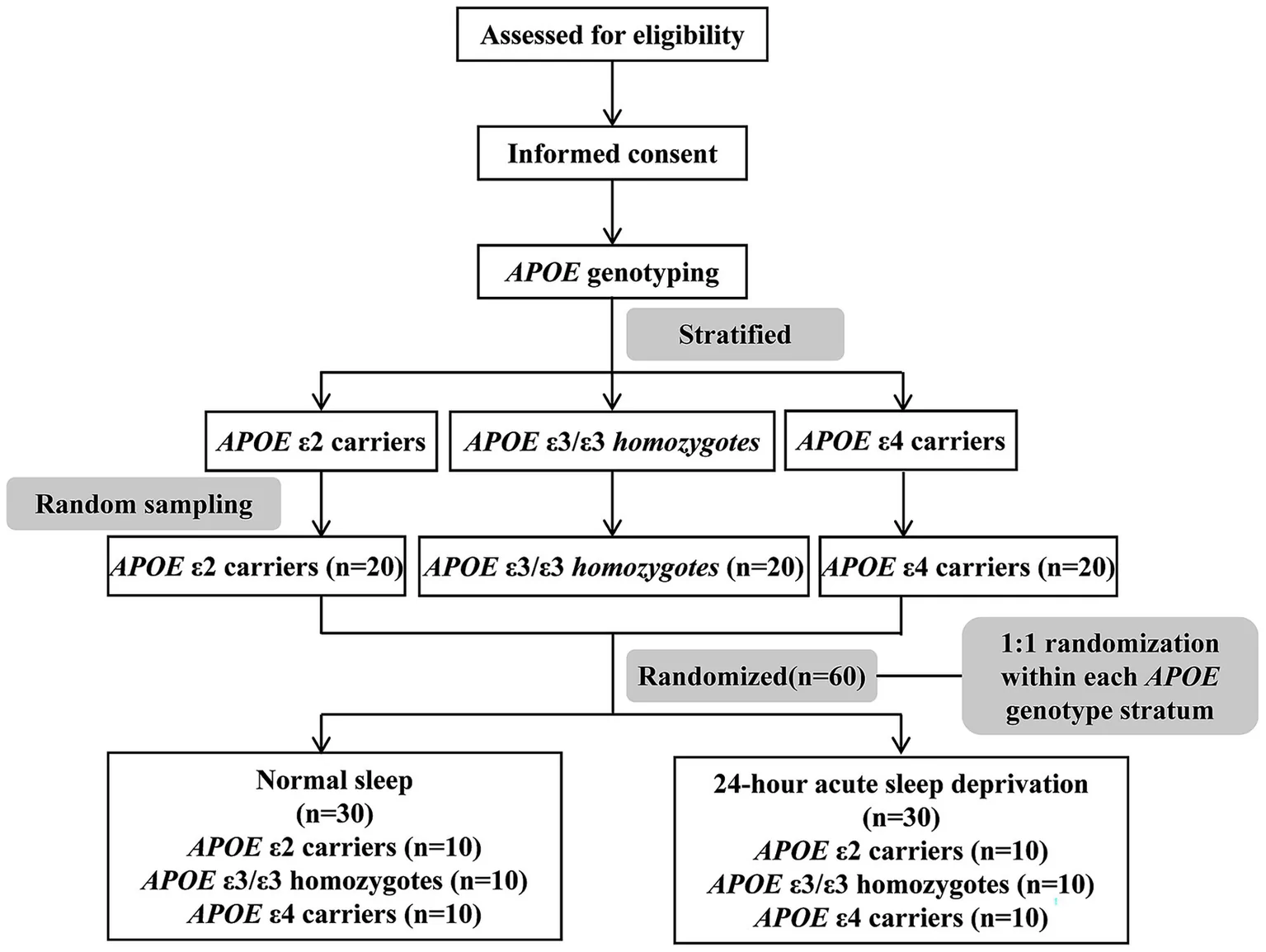
Flowchart of the trial. APOE, Apolipoprotein E.

**Table 1:** The schedule of enrollment, interventions, and assessments.

Study period timepoint	Enrolment	Pre-allocation	Allocation	Post-allocation
	−T1	T0		T1
Enrollment				
Eligibility screening	×			
Informed consent	×			
Allocation			×	
Interventions				
24-h acute sleep deprivation group			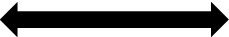	
Normal sleep group			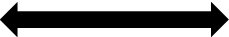	
Assessments				
Primary Outcome				
Plasma Aβ_42_ levels				×
Secondary Outcome				
Plasma Aβ_40_ levels		×		×
Plasma t-tau levels		×		×
Plasma pTau-181 levels		×		×
Plasma pTau-217 levels		×		×
Plasma NfL levels		×		×
Plasma GFAP levels		×		×
Plasma Aβ_42_/Aβ_40_levels		×		×
Plasma t-tau/Aβ_42_ levels		×		×
Plasma p-tau181/Aβ_42_ levels		×		×
Plasma p-tau217/Aβ_42_ levels		×		×
FS-14		×		×
PANAS		×		×
N-back tasks		×		×
Exploratory outcomes				
Metabolomics		×		×
Proteomics		×		×
PET/MRI		×		×

Time points: −T1, before baseline screening; T0, baseline; T1, after intervention. Aβ, Amyloid-β; FS-14, Fatigue Scale-14; GFAP, Glial Fibrillary Acidic Protein; NfL, Neurofilament Light chain; PANAS, Negative Affect Schedule; PET/MRI, Positron Emission Tomography–Magnetic Resonance Imaging; p-tau, phosphorylated tau; t-tau, total tau.

### 2.2 Recruitment and screening

Participants will be recruited through advertisements. Before screening, all potential participants will receive detailed verbal and written information regarding the study procedures, potential benefits, and risks. During the recruitment phase, we will inform all potential volunteers that, upon completion of the study, they will have the option to receive their individual APOE genotype results. This disclosure will be accompanied by professional counseling to explain the clinical implications of their genotype and to provide personalized health recommendations for the future. Recruitment and screening will proceed concurrently. Individuals expressing interest in the study will undergo screening to determine eligibility according to the predefined inclusion and exclusion criteria. Recruitment and screening will continue until the target sample size for all *APOE* genotype strata has been achieved. Recruitment will stop once the sample size target of the rarest genotype stratum is met. Subsequently, randomized sampling will be applied to the other two genotype strata to achieve the total sample size. Written informed consent will be obtained from all participants prior to participation, and participants will retain the right to withdraw from the study at any time. To maximize retention rates, identical compensation will be provided to each participant who provides written informed consent and completes the full study.

### 2.3 Eligibility criteria

Eligibility will be determined based on the following inclusion and exclusion criteria.

#### 2.3.1 Inclusion criteria

1. Aged 18–40 years, regardless of gender.
2. Healthy (with no clinically significant abnormal findings in the physical examination report or self-reporting as healthy) and not on medications.
3. Cognitively normal (Mini-Mental State Examination (MMSE) score > 28).
4. Average sleep duration of 7–9 h per night with good sleep quality (Pittsburgh Sleep Quality Index (PSQI) ≤ 5 points).
5. Written informed consent, voluntarily participate in this study, and be able to cooperate with the physician to complete the clinical study.

#### 2.3.2 Exclusion criteria

1. Presence of day-night sleep reversal.
2. Shift work within the past 6 months.
3. Travel across time zones or experience of jet lag within the past 3 weeks.
4. Current smoking or nicotine use; alcohol consumption exceeding five standard units per week [one standard alcohol unit is defined as 10 mL (or 8 g) of pure alcohol].
5. Consumption of strong tea, coffee, or caffeine-containing foods and beverages within 1 week before study participation.
6. Family history of early-onset dementia.
7. Self-Rating Depression Scale (SDS) score ≥ 53, Self-Rating Anxiety Scale (SAS) score ≥ 50.
8. Female participants who are currently pregnant or breastfeeding.
9. Individuals who need to drive or operate vehicles or machinery during the study period.

### 2.4 Randomization and blinding

The randomization sequence will be generated using SAS 9.4 by an individual statistician who will not participate in the assessment or data collection and stored in opaque envelopes. Once the envelopes have been prepared, they will be distributed to the study implementers. The study implementers will then open the envelopes in the order of participant enrolment and determine each participant’s group assignment according to the randomization sequence by genotype strata. Given the open-label design of this trial, both study implementers and patients will be aware of the treatment allocation. However, outcomes assessors within the research team and the statisticians will remain blinded to group assignments and APOE genotype status until after the final participant has completed the study and the database has been locked.

### 2.5 Interventions and control

From 08:00 to 22:00, individuals will be kept blinded to their group allocations. Quiet activities such as reading, writing, studying, working or watching a film are permitted, but vigorous exercise, highly stimulating tasks, consumption of caffeine, tea, alcohol or any medications, and napping are prohibited. Meals will be provided at 08:00, 13:00 and 19:00, and participants will have 20 min to eat each meal. The random group assignments will be revealed at 22:00.

#### 2.5.1 Intervention group: 24-h acute sleep deprivation

From 22:00 to 08:00 the following day, room lights will remain on, and illuminance will be maintained at 250 lux at eye level. Participants must remain awake in the study area and may engage in activities such as board games, watching films, or participating in group discussions. Only water consumption will be permitted and eating will be prohibited. Continuous direct visual supervision will be provided by one well-trained staff for every two participants.

#### 2.5.2 Control group: normal sleep

From 08:00 to 22:00, participants will receive the same procedures as the intervention group while remaining unaware of their group allocation. After group assignments are revealed at 22:00, participants in the normal sleep group will be permitted to complete their sleep in the laboratory and will be required to obtain approximately eight hours of sleep between 23:00 and 07:30 the following day. The participants’ sleep environment must have a light intensity of <0 lux, and their sleep will be recorded using an ActiGraph (Model: wGT3X-BT) during this period.

To standardize baseline conditions, all participants will be required to maintain and document a regular sleep–wake cycle for 1 week prior to the experimental protocol as verified by daily logs. Fasting blood samples will be collected, and all assessments will be conducted at two time points: at baseline (08.00 before randomization on day 0) and after intervention (08.00 after acute sleep deprivation/normal sleep on day 1).

### 2.6 Adverse events

Adverse events are defined as any undesirable occurrence experienced by participants during the trial period, regardless of whether it is associated with the intervention. Specific to acute sleep deprivation, particular attention will be paid to neuropsychiatric symptoms (e.g., severe irritability, confusion) and cardiovascular discomfort. Any adverse events reported by participants will be recorded by researchers during outcome measurements and intervention-related contacts. During the 24-h sleep deprivation, the intervention will be stopped immediately if the participant exhibits severe physical or mental distress, loss of consciousness, or uncontrollable hypertension. Following the occurrence of an AE, researchers must provide appropriate treatment to the participant and submit a follow-up report to the ethics committee within 15 days, detailing the cause of the event and the measures taken. Participants are informed during the consent process that while transient fatigue and mood changes may occur, no long-term adverse effects are expected from a single 24-h deprivation session.

### 2.7 Outcomes

The primary outcome is the change in plasma Aβ_42_ levels from baseline to day 1 after randomization. The secondary outcomes include changes in plasma Aβ_40_, t-tau, p-tau181, p-tau217, NfL, GFAP, and the ratios t-tau/Aβ_42_, p-tau181/Aβ_42_, p-tau217/Aβ_42_, along with changes in the Fatigue Scale-14 (FS-14), Positive and Negative Affect Schedule (PANAS), and the N-back tasks. The FS-14 is a standardized questionnaire reflecting physical and mental fatigue, comprising 14 questions. Each question has two options (yes or no), resulting in a total score ranging from 0 to 14. The first eight items comprise the physical fatigue dimension, and the last six items comprise the mental fatigue dimension. Higher scores indicate a higher level of chronic fatigue (24, 25). The Positive and Negative Affect Schedule (PANAS) consists of 20 items that assess acute emotional states. It includes 10 items measuring positive affect (PA) and 10 items measuring negative affect (NA). Participants rate each item on a 5-point Likert scale, from 1 (very mild) to 5 (very strong) (26). The two N-back tasks (1-back and 2-back) will be used to assess working memory. In the 1-back task, participants will be instructed to indicate whether the current number matches the number presented on the immediately preceding screen. In the 2-back task, participants will indicate whether the current number matches the number presented two screens earlier. Accuracy rates will be calculated for each task (27).

Exploratory outcomes will include changes identified through plasma proteomics and, metabolomics, as well as positron emission tomography/magnetic resonance imaging (PET/MRI).

### 2.8 Study arrangement

On the day of the intervention, all participants will be required to arrive at the study area at 08:00 after an overnight fast for collection of the first blood sample and completion of all baseline assessments. Demographic data to be collected will include age, sex, education level, and Body Mass Index (BMI), the schedule of assessments and study procedures for all participants is presented in Table 1. A second fasting blood sample will be collected, and other assessments will be conducted at 8:00 on the following day.

### 2.9 Sample size

Given the limited number of clinical studies examining the effects of sleep deprivation on AD biomarkers in humans, this study is designed as an exploratory trial and plans to enroll a total of 60 participants.

### 2.10 Data collection, management and monitoring

Data collection for the study will take place during the baseline phase and on Day 1 following randomization. All assessors will receive adequate training prior to conducting the study. If the participant withdraws consent from participating in the study, no further evaluations will be performed, and no additional data will be collected. This study will be conducted in compliance with Good Clinical Practice (GCP). The Data Manager will oversee all data processes, including the design of Case Report Forms (CRFs) and Standard Operating Procedures (SOPs) established prior to data collection. Investigators and coordinators are required to complete CRFs accurately and promptly. Any data queries (DRQs) will be managed by the Data Manager via monitors, with investigators mandated to respond in a timely manner. All data, including audit trails and management logs, will be retained in full. Personal data of the participants will be treated as confidential and stored securely during the whole course of this trial.

### 2.11 Blood sample collection

For both blood collections, five professional nurses will be assembled to begin phlebotomy simultaneously for all participants, ensuring that the entire collection process is completed within 15 min to avoid the influence of chronological time on individual plasma biomarker concentrations. All samples will be collected in ethylenediaminetetraacetic acid (EDTA) tubes at a standardized time point to minimize the influence of circadian rhythms. Plasma will obtained by centrifugation (4 °C, 1800 g, 10 min) within 1 h of collection. Following collection and labeling of the two plasma samples, they were stored at −80 °C.

### 2.12 AD biomarkers

After the completion of plasma sample collection for all AD biomarkers requiring analysis, the frozen plasma samples will be thawed to room temperature, vortexed, and centrifuged at 10,000 × g for 10 min to remove any particulates prior to analysis. Subsequently, the concentrations of Aβ42, Aβ40, GFAP, NfL, p-tau217, p-tau181, and t-tau will be quantified using Single Molecule Array (Simoa) technology on a fully automated HD-X Analyzer (Quanterix, Billerica, MA, USA). All measurements will be performed using commercially available kits. NfL, GFAP, Aβ42, and Aβ40 will be measured using the Simoa^®^ Human Neurology 4-Plex E (N4PE) PLUS kit. The p-tau181 levels will be assessed with the Simoa^®^ p-Tau 181 Advantage V2.1 kit, the p-tau217 levels will be measured using the Simoa^®^ p-Tau 217 PLUS kit, and the t-tau levels will be measured using the Simoa^®^ Tau 2.0 kit. Prior to analysis, it is imperative to ensure that both pre- and post-intervention plasma samples from the same participant are assayed on the same plate to avoid inter-plate variation. All analyses will be performed by qualified technicians who are blinded to the clinical data. Detailed standardized procedures will be followed throughout the analysis to ensure analytical precision.

### 2.13 Statistical analysis

The primary analyses will be conducted on the modified intent-to-treat (ITT) population, defined as all randomized participants who have undergone assessments of AD biomarkers. Participants in the control group with actigraphy-derived sleep efficiency <80% will be excluded from the primary analysis to ensure comparisons are restricted to individuals with adequate sleep quality. Baseline demographic and clinical characteristics will be summarized according to the randomized groups. Continuous variables will be presented as mean ± standard deviation or median (inter-quartile range, IQR) and compared using Student’s *t*-test or Wilcoxon rank-sum test; categorical variables will be described as frequency (%) and analyzed via *χ*^2^ test, Fisher’s exact test and Wilcoxon rank-sum test.

The effects of the intervention on all primary and secondary outcomes will be analyzed using generalized linear mixed models (GLMMs), with adjustments of age, sex, educational level, BMI, and baseline level of the corresponding outcome measures. To further examine whether the *APOE* genotype moderates the relationship between the intervention and the outcomes, we will incorporate the *APOE* genotype × intervention interaction term as an additional independent variable.

Following preprocessing, intergroup differences in both the multi-omics and PET/MRI datasets will be analyzed using Student’s *t*-tests or Wilcoxon signed-rank tests, as appropriate. This will be followed by Pearson’s correlation analysis to evaluate the associations between these variables and changes in AD biomarkers. If the proportion of missing data exceeds 10%, multiple imputation techniques will be employed to ensure the robustness of our study results. The test on the primary outcome will be based on a 95% confidence interval with a two-sided significance level of *p* ≤ 0.05. Further analyses of other outcomes and pre-specified subgroup analyses by APOE genotypes will not be adjusted for multiple comparisons and should not be interpreted as definitive evidence of treatment effects. All analyses will be performed using SAS 9.4.

### 2.14 Patient and public involvement

Patients or the public were not involved in the design of our research. The results will be disseminated to patients in a lay summary format.

## 3 Discussion

To our knowledge, this study will be the largest randomized controlled trial (RCT) to investigate the effects of acute sleep deprivation on healthy young individuals, and firstly combined with *APOE* genotype stratification. A multidimensional phenotyping strategy encompassing plasma AD biomarkers, neuropsychiatric and cognitive assessments, multi-omics, and neuroimaging will be employed to investigate the 24-h acute sleep deprivation on pathophysiology of AD.

This study has several advantages. First, it uses quantifiable biomarkers to reveal the effects of acute sleep deprivation on healthy young individuals. Second, by including individuals with different *APOE* genotypes, the study explores genetic susceptibility to AD induced by acute sleep deprivation. Third, by applying peripheral blood multi-omics analysis (proteomics and metabolomics), the study can investigate the molecular responses triggered by acute sleep deprivation at a systemic level and pinpoint potential circulating biomarkers. Fourth, the findings hold particular clinical relevance for populations frequently exposed to sleep loss, such as shift workers, healthcare professionals, and emergency responders. Although acute changes cannot directly predict long-term outcomes, demonstrating that even brief sleep deprivation significantly alters core AD biomarkers provides a mechanistic basis for understanding how chronic sleep disruption may accumulate to increase dementia risk in these vulnerable groups. Finally, by characterizing the immediate molecular consequences of total sleep deprivation, this study establishes a crucial foundation for future investigations aimed at elucidating the link between chronic sleep deficiency and long-term neurodegenerative diseases.

In summary, this work will provide comprehensive insights into the effects of sleep deprivation on AD biomarkers and offer novel evidence to inform the rationale for early sleep interventions in populations with differential genetic risks for AD.
